# Introductory Addresses

**Published:** 1868-12-15

**Authors:** 


*Introductory Addresses*

By E. B. Stevens, M.D., Theoph. Parvin, M.D., and J. Aitken Meigs, M.D., are on file for notice. We have only space here to say that Dr. Stevens' address before the Ohio State Medical Society, and his Introductory to the College session, are productions of the highest order, and sufficient alone to establish a wide reputation for their accomplished author.

